# Hematopoietic stem cells have an intrinsic expansion limit

**DOI:** 10.1186/1753-6561-7-S2-O6

**Published:** 2013-04-04

**Authors:** Shanti Rojas-Sutterlin, André Haman, Trang Hoang

**Affiliations:** 1 Institute for Research in Immunology and Cancer (IRIC), Université de Montréal, Montréal, Canada

## Background

Hematopoietic stem cell (HSC) transplantation is the only treatment providing long-term cure in acute myeloblastic leukemia. At the apex of the hematopoietic system, quiescent HSCs escape to chemotherapeutic treatments and therefore can regenerate the entire blood system after drug exposure. Nevertheless, the consequence of repeated chemotherapy regimen on HSC function remains to be clarified. In this study, we investigate how massive expansion *in vivo* influence HSC functions.

## Methods and results

We optimized a protocol based on 5-fluorouracil (5FU), an antimetabolite used to treat different types of cancers. We show that after one 5FU treatment, HSCs exit quiescence and enter the cell cycle. To deplete cycling HSCs, we injected a second dose of 5FU and show that the stem cell pool is disseminated. Nonetheless, the remaining HSCs proliferate extensively to re-establish the HSC pool. At this point, most HSCs have exited the cell cycle and are back to quiescence. Despite a near normal stem cell pool size and a quiescent status, HSCs from these 5FU treated mice cannot compete against untreated cells to regenerate the host in transplantation assays. Furthermore, we show that this extensive proliferation *in vivo* severely impairs the clonal expansion of individual HSC as measured by the mean activity of stem cell (MAS).

## Conclusion

Our results demonstrate that HSCs loose their competitive potential after two 5FU treatments, suggesting that HSCs have an intrinsic expansion limit beyond which their regenerative potential is impaired. We surmise that chemotherapy regimens based on repeated administration of antimetabolites are likely to impair long-term stem cell functions.

## Financial support

FRSQ, Cole Foundation.

